# Bond strength of self-adhesive resin cement to dentin using different adhesion protocols

**DOI:** 10.4317/jced.59043

**Published:** 2022-01-01

**Authors:** Luiz-Roberto-Dallari Junior, Kusai Baroudi, Leonardo-dos Santos Barroso, Milton-Edson Miranda, Rafael-Pino Vitti, William-Cunha Brandt

**Affiliations:** 1 School of Dentistry, University of Sao Leopoldo Mandic, Campinas, Sao Paulo, Brazil; 2 Postgraduate Program, School of Dentistry, University of Taubaté, Taubaté, Sao Paulo, Brazil; 3 School of Dentistry, University Center of Herminio Ometto Fund, Araras, Sao Paulo, Brazil; 4 School of Dentistry, University of Santo Amaro, Sao Paulo, Brazil

## Abstract

**Background:**

The treatment of dentin before the use of self-adhesive cements is still a crucial point to achieve the best bond strength values. The objective of this study was to evaluate the bond strength between dentin and composite resin using different adhesion strategies with self-adhesive resin cement.

**Material and Methods:**

Forty healthy third human molars were randomly divided into 4 groups (n = 10): CA (control); application of self-adhesive cement (Rely X U200, 3M ESPE), AD + CA: only application of conventional adhesive (Adper Single Bond 2, 3M ESPE) + self-adhesive cement, AC + AD + CA; conditioning with 37% phosphoric acid for 15 seconds + application of conventional adhesive + self-adhesive cement and AC + CA; conditioning with 37% phosphoric acid for 15s + self-adhesive cement. Blocks made of composite resin (Z250 XT, 3M ESPE) were cemented over dentin. The samples were stored for 24h in distilled water at 37ºC and then were sectioned on a metallographic cutter to obtain tooth picks with approximately 1.0 mm2 in cross section. A universal testing machine was used with a speed of 0.5 mm/min to test the microtensile bond strength,. The fracture patterns were classified as adhesive, cohesive and mixed failures. The data (MPa) were analyzed statistically by One-way ANOVA and Holm-Sidak test (α=5%).

**Results:**

The AC + AD + CA and AC + CA groups had the highest averages, while the CA and AD + CA groups had the lowest bond strength values. Adhesive failure was prevalent in all groups.

**Conclusions:**

Conditioning with 37% phosphoric acid for 15s increases the adhesion of the self-adhesive resin cement to the dentin, regardless of the use of dental adhesive system.

** Key words:**Resin cement, microtensile bond strength, acid conditioning.

## Introduction

Self-adhesive cements, by simplifying the number of steps in the technique, make the cementation procedure less sensitive to errors, unlike what happens with conventional cements, with more clinical stages (1-5). Another improvement arising from this material is the dual activation system (1-3,6), ie, polymerization begins with a chemical reaction that requires photoactivation to complement the final setting of the material.

The manufacturer’s recommendation does not require the prior stage of conditioning the dental substrates, and washing with application of primer and adhesive, as self-adhesive cements are capable of modifying the smear layer and incorporating it into the hybrid layer (7-10). This is because one of its components, the multifunctional acid methacrylate (carboxylic or phosphoric) (11), has the ability to demineralize the dental substrate, due to its acidity, mainly facilitating the penetration of the resinous component of the cement into the matrix dentin (8). Adhesion to these materials involves the initial action of acidic monomers on enamel and dentin, which simultaneously promote demineralization and infiltration of the cementing agent into the conditioned area, resulting in micromechanical retention (1-3). Secondary reactions, also important, occur between the self-adhesive cement and hydroxyapatite, through chemical bonds with Calcium ions (12).

However, despite all the positive characteristics disclosed for self-adhesive cements, some studies (13,14) cite lower bond strength values for this material. Regarding enamel substrate conditioning, Moghaddas *et al*. (4) mentioned that there is no influence on the strength of the union. However, for the dentin substrate, it is argued that the self-adhesive cement is not able to demineralize/dissolve the smear layer completely. And this partial demineralization of the smear layer results in a lower potential for adhesion to dentin (4,13,14).

The big question about the use of self-adhesive cements is still about the previous treatment of dentin. Some studies suggest that cement alone is capable of demineralizing and incorporating the smear layer in the dentin bonding process (1-3). Others indicate that maintaining the smear layer can interfere with the adhesive efficiency of such materials (4,13,14). But the fact of conditioning the dentin can also hinder the adaptation and adhesion of the resin material, as it increases the dentin water flow, and can compromise the adhesive bond, in addition to interfering with the polymerization of the material (15).

Thus, the aim of the present study was to evaluate the bond strength, through a microtensile test, using different protocols for bonding to dentin with a self-adhesive resin cement. The hypothesis of the present study is that the different adhesive protocols influence the adhesion of dentin to the self-adhesive resin cement.

## Material and Methods

Forty healthy third human molars were selected (approved by the ethical committee of the University of Sao Leopoldo Mandic, Brazil, CAAE:4355115.9.0000.5374), extracted and preserved in 0.1% Timol at 4ºC, were sectioned in the occlusal third of the crown, in the mesio-distal direction, perpendicularly along the long axis of the tooth and another cut parallel to this was made in the third of the crown exposing the dentin fully. Pumice stone prophylaxis was performed on the surfaces and the cut teeth were washed and kept in distilled water at 37°C in an oven.

They were randomly divided into 4 groups (n = 10);

Group CA (control): application of self-adhesive cement Rely-X U200 only following the manufacturer’s recommendations;

Group AD + CA: application of Adper Single Bond 2 adhesive (3M do Brazil, Sumaré, Brazil) on the dentin surface with microbrush, photopolymerized for 30 seconds, prior to the application of the self-adhesive cement Rely-X U200;

Group AC + AD + CA: conditioning with 37% phosphoric acid for 15s (Condac 37%, Dentscare, Joinville, Brazil)flushing with running water from the triple syringe for 30s, lightly drying the dentin surface with a cotton ball, in order to maintain a slight shine on the surface, applying the Adper Single Bond 2 adhesive prior to the application of the Rely-X U200 self-adhesive cement;

Group AC + CA: conditioning with 37% phosphoric acid for 15s and application of self-adhesive cement Rely-X U200.

After the application of the resin cement, blocks made of light-cured resin (Z-250 XT 3M / ESPE, Irvine, USA) were cemented over the dentin. The photoactivation of the resin cement was carried out by a Radii photopolymerizer (SDI, Bayswater, Australia), with LED application for 60s, with 1200 mW/cm². Then, the samples were stored for 24 hours in distilled water at 37°C.

After that time, the samples were sectioned in a metallographic cutter (Isomet) to obtain toothpicks with approximately 1.0 mm² of cross section. For the microtensile test, a universal testing machine (EMIC - DL2000) with a speed of 0.5 mm/min was used. The failure pattern was analyzed under an optical microscope and classified into adhesive, cohesive and mixed failures. Prior to the analyzes, fracture resistance data were assessed for normality using the Kolmogorov-Smirnov test. The data (MPa) were analyzed statistically by One-way ANOVA and Holm-Sidak test (5%).

## Results

Analysis of variance at one criterion showed that there was a statistically significant difference in the strength values of the Bonding between the protocols used. According to the Holm Sidak test, AC + AD + CA and AC + CA demonstrated the highest bond strength values, which did not differ. While AD + CA and CA showed the lowest values of bond strength with no difference between them.

**Figure 1:**
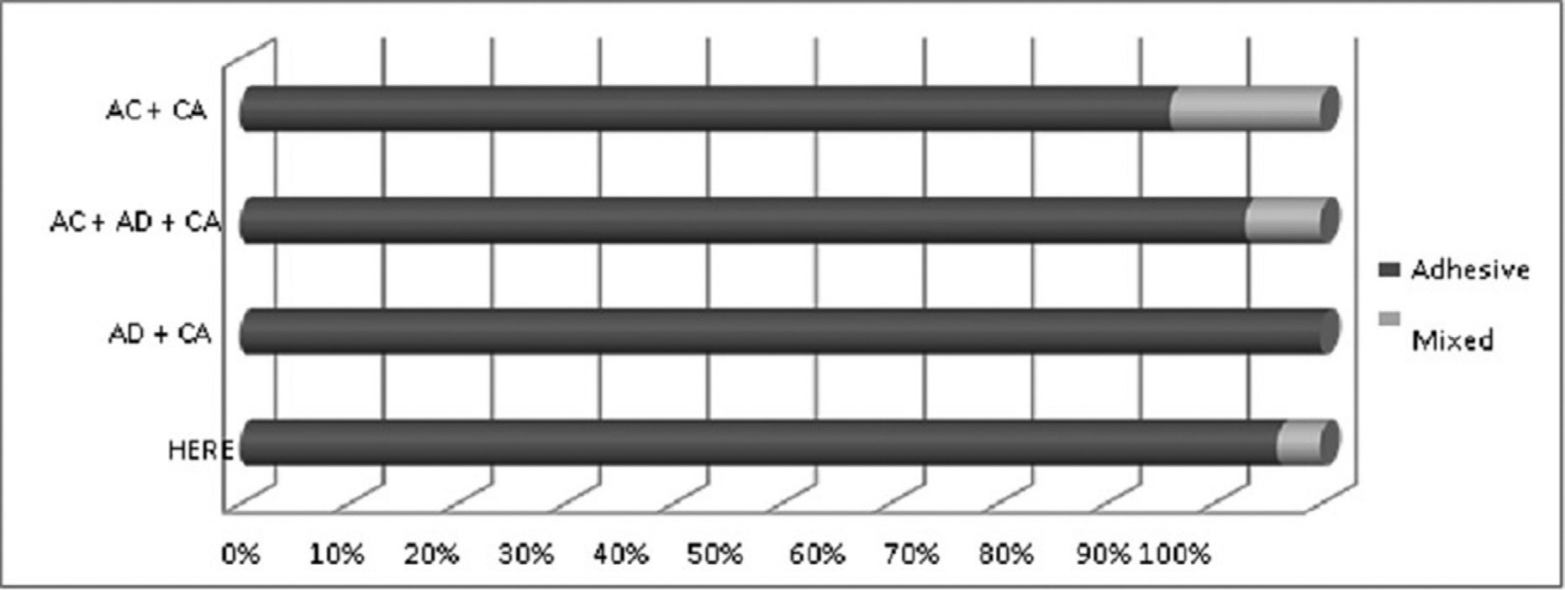
Types of failure percentage of types of failure that occurred in the different protocols.

The predominant failure pattern among all evaluated techniques was the adhesive type failure. AD + CA obtained the highest number of adhesive failures (100%) and AC + CA the lowest (86%) (Fig. 1, Table 1).

**Table 1:**
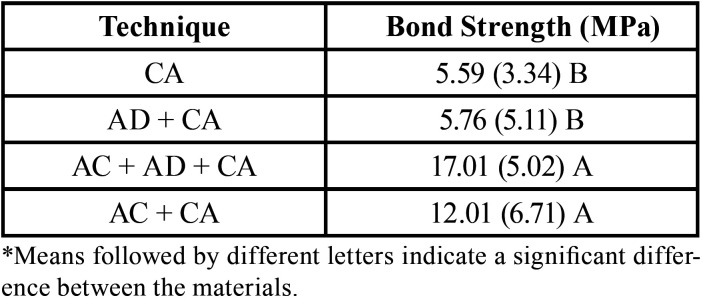
Mean and standard deviation of the bond strength values of the dentin resin cement bond.

## Discussion

The main objective of the cementation procedure is to establish a stable bonding between the tooth structure, the restorative material and the cementation agent itself. Although some authors cite that self-adhesive cements have greater bond strength than conventional systems (16), other studies cite self-adhesive cements as still inferior to conventional cements, due to the fact that, to improve the strength of bonding, require the application of adhesive systems before cementation (17,18).

For Moghaddas *et al*. (4) the RelyX U200 cement, also used in this research, has the advantage of containing methacrylate monomers in the base paste along with acid phosphoric groups. These monomers have a self-etching action on the tooth surface, through the ionization reaction of the acid monomer with the water present on the enamel and dentin surfaces. Based on research by De Munk *et al*. (8) water is necessary for the ionization of the acid present in the composition of the RelyX U200 cement and contributes to a “corrosive efficiency of the monomer”. The catalyst slurry of this material also has more alkaline compounds. Thus, all material reacts with the hydroxyapatite of enamel and dentin, while neutralizing the cement composition (18). The micromechanical retention and chemical interaction of acidic monomers containing phosphate group with the hydroxyapatite of the tooth lead to the desirable bond strength for such a resin cement (19).

However, other authors claim that RelyX U200, due to its low pH values and, consequently, of interaction with the tooth surface, contributes to the formation of a hybrid layer with questionable quality (20,21). What is discussed in the literature is that with the use of self-adhesive cements, the incomplete penetration of resin monomers into the dentin collagen network reduces the quality of the hybrid layer (22).

In the present study, the resin cement RelyX U200 obtained the highest bond strength values when the dentin was previously etched with 37% phosphoric acid, with or without the subsequent application of the adhesive. The dentin etching procedure aimed to remove the smear layer, in order to open the dentinal tubules and expose the collagen fibers, improving the wetting capacity and, consequently, the penetration of the adhesive or cement system on the fibers of collagen. Thus, a better micromechanical retention mechanism is established, which was proven through the results for the groups AC + AD + CA and AC + CA, which obtained the highest values of bond strength.

Some authors emphasize better bond strength values, from dentin to self-adhesive cement, when using polyacrylic acid to treat the dentin surface. The justification is due to the fact that RelyX U200 contains a mixture of monomers and phosphoric acid associated with glass ionomer particles, having similar characteristics to glass ionomer cement (5). However, the same authors mentioned that, even in an immediate analysis, the dentin bond strength was more efficient with previous dentin treatment, after 6 months of sample storage; statistically different results were not observed compared to the control group (no previous treatment). In other words, in the long term, the treatment of the dentin surface would not have negative or positive effects in terms of bond strength. Other author (4) pointed out that the acid etching process in dentin with phosphoric acid before the application of the self-adhesive cement reduces bond strength, as the synergistic effect of phosphate monomers of RelyX U200 cement, together with acid phosphoric, cause dentin hyper demineralization with damage to peritubular and intertubular dentin.

The positive results of the present study for improving bond strength can be explained by the fact that the phosphate group of the monomers of the RelyX U200 cement has the ability to bind, in a very intense and sTable way, to the hydroxyapatite of dentin and enamel, more precisely to calcium ions, forming MDP-Ca, which increased the success of the technique and, consequently, improves the longevity of the restorations (23,24). As mentioned in the literature, the lower adhesive efficiency to dentin and the higher prevalence of adhesive failures between resin cement and dentin were detected when using self-etching resin cement used as recommended by the manufacturer, without prior conditioning of the substrate (23-25).

It is noteworthy that, in relation to the fractures of the specimens obtained in the present study, it was possible to observe fractures at the adhesive interfaces between dentin/resin cement and dentin/resin block, corroborating other studies that mention the methodological effectiveness of research on bond strength when fractures occur in prevalence at the adhesive interface (26,27).

The longevity of the restoration depends on the numerous clinical steps prior to the actual restorative procedure. Of course, the use of good materials is essential for the success of a restorative procedure, always with the objective of guaranteeing an improvement in the bonding of the restorative material to the dental tissues. But, for clinical success to occur, regardless of the material used, it is extremely important that the professional has dexterity and care with the technique to be performed. Even the form of insertion of the material must avoid the formation of failure in the continuity and incorporation of bubbles, for example. The diameter and thickness of the cement is also well discussed regarding its influence on the clinical outcome, although there is still no consensus on the ideal thickness of the resin cement interface (15). These are some of the factors that can affect bond strength tests.

Through this methodological laboratory analysis, it is possible to affirm that the standardization of the steps of the cementation process, together with the results obtained, suggests that the application of phosphoric acid (37%) positively influences the adhesion of the cementation process of self-adhesive materials. However, because there are still contradictions in the literature on the subject, self-adhesive cementation and its protocols need more research, both in the laboratory and, especially, in clinical trials.

## Conclusions

Etching with 37% phosphoric acid for 15s increases the adhesion of the self-adhesive resin cement to the dentin substrate, regardless of the use of an intermediate adhesive system.
